# Single-Molecule Three-Color FRET with Both Negligible Spectral Overlap and Long Observation Time

**DOI:** 10.1371/journal.pone.0012270

**Published:** 2010-08-19

**Authors:** Sanghwa Lee, Jinwoo Lee, Sungchul Hohng

**Affiliations:** 1 Department of Physics and Astronomy, Seoul National University, Seoul, Korea; 2 Department of Biophysics and Chemical Biology, Seoul National University, Seoul, Korea; 3 National Center for Creative Research Initiatives, Seoul National University, Seoul, Korea; University Paris 7, France

## Abstract

Full understanding of complex biological interactions frequently requires multi-color detection capability in doing single-molecule fluorescence resonance energy transfer (FRET) experiments. Existing single-molecule three-color FRET techniques, however, suffer from severe photobleaching of Alexa 488, or its alternative dyes, and have been limitedly used for kinetics studies. In this work, we developed a single-molecule three-color FRET technique based on the Cy3-Cy5-Cy7 dye trio, thus providing enhanced observation time and improved data quality. Because the absorption spectra of three fluorophores are well separated, real-time monitoring of three FRET efficiencies was possible by incorporating the alternating laser excitation (ALEX) technique both in confocal microscopy and in total-internal-reflection fluorescence (TIRF) microscopy.

## Introduction

Many fundamental processes in biology occur in nanometer scale, a range in which conventional optical microscopy does not work. Single-molecule fluorescence resonance energy transfer (FRET) emerged as a tool for studying molecular interactions and dynamics with sub-nanometer sensitivity in unprecedented detail [1], [2]. Due to its unique flexibility and adaptability, single-molecule FRET is rapidly expanding its application area in biological studies. As our research interest turns into more realistic situations inside the cell, however, interactions tend to become more complex, and thus the capability of acquiring multi-dimensional information is also required. To cope with the challenge, researchers are trying to develop more versatile single-molecule FRET techniques, by combining FRET with optical tweezers [3], [4], or magnetic tweezers [5], [6]. Adding multi-color capabilities to single-molecule FRET experiments is on the same line of technical development.

Compared to the conventional single-molecule two-color FRET, multi-color FRET technique is expected to provide correct information of multi-domain configurations and their correlated motion. For the last five years, several groups succeeded in developing different versions of single-molecule three-color FRET technique [7]–[10], and important technical advances were achieved. For example, fast switching of multiple excitation lasers, called ALEX (Alternating Laser EXcitation) technique [11], [12], enabled us to determine both correct stoichiometry and multiple FRET efficiencies in real time [9]. Replacement of conventional beam splitters with a programmable acousto-optic beam splitter increased the detection yield by 10∼30% by reducing the number of optics required for multicolor imaging [10].

Despite these impressive technical advances, existing techniques still have much room for improvement. For example, relatively severe photobleaching of Alexa 488 or its alternative dyes has limited the total observation time [13], [14]. Thus, single-molecule three-color FRET technique has been limitedly used for reaction kinetics studies. Considering that kinetics information is critical in fully understanding biochemical reactions, it is required to develop a new single-molecule three-color FRET technique with long observation time comparable with single-molecule two-color FRET.

In this study, we developed a single-molecule three-color FRET technique based on cyanine dyes– Cy3, Cy5, and Cy7. By using the conventional oxygen scavenging system with Trolox, photobleaching of all fluorophores could be greatly reduced [15]. Well-separated emission peaks of the three fluorophores made data analysis and data interpretation more reliable. Real-time monitoring of multiple FRET efficiencies could be realized by using alternating laser excitation (ALEX) technique both in confocal microscopy and in total-internal-reflection fluorescence (TIRF) microscopy.

## Materials and Methods

### Fluorophores and setup

**Figure 1:**
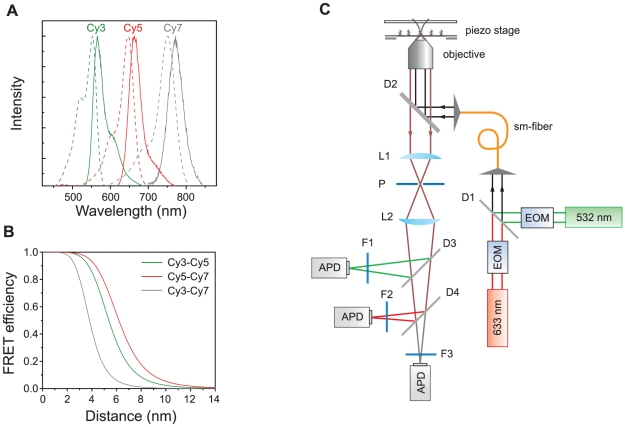
Dye selection and a confocal setup. (a) Normalized emission (solid lines) and absorption (dash lines) spectra of Cy3 (green), Cy5 (red) and Cy7 (gray). (b) FRET efficiencies of the three FRET pairs as a function of inter-dye distances. R_0_ values of Cy3-Cy5 (green), Cy5-Cy7 (red) and Cy3-Cy7 (gray) pairs were calculated as 5.4-nm, 6.2-nm, and 3.8-nm, respectively. (c) A schematic diagram of ALEX three-color confocal setup. The setup was built based on an inverted microscope (TE2000-U, Nikon, Tokyo, Japan) equipped with a three dimensional piezo-stage (LP-100, MadCityLabs, Madison, WI). Two excitation lasers, a diode-pump solid state laser (532-nm, Excelsior-CDRH, Spectra-Physics, Santa Clara, CA) and HeNe laser (633-nm, HRP050, Thorlabs, Newton, NJ), were alternatively switched on and off by using electro-optic modulators (EOM, 350-50, Conoptics, Danbury, CT). To make sure that the two excitation lasers excite the same molecule, they were coupled into a single-mode fiber (460HP, Thorlabs). An oil-immersion objective (UPLSAPO 100×, Olympus, Tokyo, Japan) was used for both the excitation of molecules and the collection of fluorescence signals. The fluorescence signals are measured by using avalanche photo diodes (APD, SPCM-AQRH-14, Perkin Elmer, Wellesley, MA). The identities of other optics are: D1, a dichroic mirror (z532bcm, Chroma, Rockingham, VT); D2, dichroic mirror (z532/633rpc, Chroma); P, pinhole (P75S, Thorlabs); L1 and L2, lens (LAO-90.0-25.0/078, CVI, Irvine, CA); D3, dichroic mirror (640dcxr, Chroma); D4, dichroic mirror (740dcxr, Chroma); F1, bandpass filter (HQ580/60m-2p, Chroma); F2, bandpass filter (HQ680/60m-2p, Chroma); F3, bandpass filter (HQ790/80m, Chroma).

Due to their superior brightness, and photostability, cyanine dyes are popularly used in single-molecule FRET experiments [14]. Their photostability can be even further improved by removing ambient oxygen and adding reducing agents [15]. From these considerations, we selected Cy3, Cy5, and Cy7 as fluorophores in this work. The choice is a significant improvement from the previous dye trio–Cy3, Cy5, and Cy5.5 [7] because huge spectral overlap between Cy5 and Cy5.5 can be avoided in the new dye trio (Figure 1a). Due to this negligible spectral overlap, data analysis became clearer and more reliable, making it possible in many cases to qualitatively understand molecular motion even without data correction steps.

Initially we were concerned about a short working range of the Cy3-Cy7 FRET pair. To compare the FRET ranges of the three FRET pairs, a Förster distance, R_0_, for each FRET pair was calculated from the following equation: R_0_^6^ = 0.529 κ^2^ Φ_D_ J(λ)/N_A_n^4^ [16], where R_0_ and λ are in the unit of centimeter, Φ_D_ is the donor quantum yield, J(λ) is the spectral overlap of the donor emission and the acceptor absorption, N_A_ is the Avogadro number, n is the refractive index of the medium, and κ^2^ is determined by the relative orientation of the two dyes. By using the quantum yields of Cy3 and Cy5 provided by the GE Health care (16% for Cy3, and 27% for Cy5), assuming the κ^2^ is 2/3, and calculating J(λ) from the measurements, Förster distances of Cy3-Cy5, Cy5-Cy7, and Cy3-Cy7 FRET pairs were obtained as 5.4-nm, 6.2-nm, and 3.8-nm, respectively. Figure 1b shows FRET efficiencies of the three FRET pairs as a function of inter-dye distances, based on the above calculation. Even though the emission spectra of Cy3 and Cy7 are almost completely separated, the graph shows that the Cy3-Cy7 pair is a good FRET probe in the range of 2-nm to 5-nm.

The well-separated absorption spectra of the new dye trio enabled us to incorporate the ALEX technique, which was not feasible in the previous Cy3-Cy5-Cy5.5 dye trio. To realize the ALEX technique in confocal microscopy (Figure 1c), we followed the scheme of Namki Lee *et al* [9]. Electro-optic modulators were used to alternatively switch a green laser and a red laser. To make easier the colocalization of the excitation lasers, they were coupled into the same single mode fiber. We used three avalanche photodiodes as a fluorescence detector. Details of optics are described in the figure caption. Data acquisition of fluorescence signals were synchronized with the laser switching by using a common trigger signal of a timer/counting board (DAQ PCI-6602, NI, Austin, TX). The whole setup was run by a home-made program.

### Correction of background, bleedthrough, and direct excitation

Before FRET analysis, all data were corrected for background noise, bleedthrough between detection channels, and direct excitation of acceptors as follows. To generalize the following discussion, we use indices to indicate FRET probes and detection channels in the increasing order of emission peaks. So, in this paper, the first, the second, and the third dyes correspond to Cy3, Cy5, and Cy7, respectively. In the discussion of this section, we don't specify which excitation laser is used, because it does not affect the discussion. Thus the following symbols are:

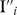
, total fluorescence intensity in the i^th^ detection channel after background subtraction

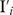
, fluorescence intensity of the i^th^ dye detected in the i^th^ detection channel

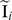
, sum of fluorescence intensities of the i^th^ dye detected in the all three channels.

The first data correction step was background subtraction. Next we corrected the bleedthrough between detection channels. By assuming no bleedthrough from the longer wavelength dyes into the shorter wavelength detection channels, we get the following expressions.
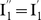
(1)

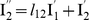
(2)

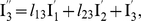
(3)where l_ij_ is the bleedthrough parameter of the i^th^ dye into the j^th^ detection channel. For example, 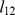 denotes the amount of Cy3 fluorescence signal detected in the second detection channel in units of Cy3 signal detected in the first detection channel. Specifically, 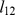, and 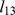 can be determined from the ratios of 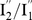, and 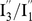 when only Cy3 is present. Similarly, 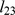 is the ratio of 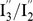 when only Cy5 is present. By solving the above equations, we obtained the following equations.
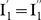
(4)

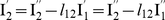
(5)

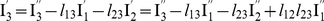
(6)


Next, we obtained 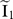, 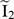 and 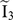 by adding back the bleedthrough signals into the proper detection channels as follows.
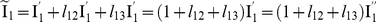
(7)

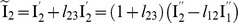
(8)

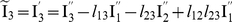
(9)


To correctly calculate the FRET efficiency of each FRET pair, we should consider the differences in emission quantum yields of fluorophores, and the detection efficiencies of the detection channels, which can be corrected as follows, a step called the gamma correction [2], [14].
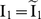
(10)

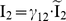
(11)

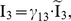
(12)where 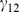 is the ratio of 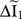 to 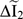, and 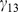 is the ratio of 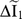 to 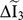 during a conformational change or photobleaching. Because these gamma factors vary depending on dye labeling scheme and optical design, they should be determined case by case. Bleedthrough and gamma correction parameters used in our experiment are summarized in Table 1.

**Table 1:** The correction parameters used in this work.

Parameters	Confocal setup	TIRF setup
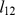	0.13	0.13
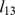	∼0	∼0
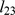	0.11	0.18
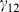	HJ: 0.67, Dp: 0.88	HJ: 0.60
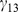	HJ: 1.28, Dp: 1.97	HJ: 1.50

The identities of symbols are defined in the text. The gamma correction parameters are for the Holliday junction (HJ), and for DNA duplexes (Dp).

In FRET experiments, it is ideal for each laser to excite only one fluorophore, which is a good approximation for green laser excitation as the absorption of Cy5, and Cy7 at 532-nm are just 3% and 2% of their peak values, respectively. However, the absorption of Cy7 at 633-nm is not negligible (13% of its peak value), and thus 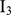 at red excitation condition is contributed by both FRET from Cy5 and the direct absorption of the excitation laser. Fortunately the direct excitation part can be removed as follows. We assume that the ratio of the direct absorption of Cy7 to the absorption of Cy5 is conserved, and thus the ratio of Cy7 fluorescence due to the direct absorption to the sum of I_2_ and I_3_. Because the Cy7 fluorescence when Cy5 is photobleached comes only from the direct excitation of Cy7, we can obtain the portion of the direct excitation of Cy7 in I_2_ + I_3_ by comparing I_2_ + I_3_ values before and after Cy5 bleaching. By analyzing intensity time traces of ∼20 molecules, we concluded that 20% of I_2_ + I_3_ come from the direct excitation of Cy7, a number similar to the ratio of Cy7 absorption to that of Cy5 at 633-nm (19%). From now on, we will use I_3_ to denote Cy7 intensity after the subtraction of the direct excitation contribution.

### DNA preparation

To test our ALEX single-molecule three-color FRET setup, we used two DNA constructs: a DNA duplex and the Holliday junction. To assemble DNA duplexes, we purchased the following DNA sequences (written from 5′ to 3′) from IDTDNA (Coralville, IA).

**a**: biotin-CCGTA**T**GTAGCAACAGAGCGGTGGG

**b_1_**: Cy5-CCCACCGCTCTGT**T**GCTACATACGG

**b_2_**: Cy5-CCCACCGCTCTG**T**TGCTACATACGG

**b_3_**: Cy5-CCCACCGCTC**T**GTTGCTACATACGG

The bold-faced T in the strand **a** was internally labeled with Cy7, and those in the strand **b**'s with Cy3. By annealing the strand **a** and **b_1_**, DNA duplex dp1 was made. In the same way, dp2 and dp3 were made by annealing **a** with **b_2_** or **b_3_**, respectively.

To construct the Holliday junction, the following DNA sequences (written from 5′ to 3′) were purchased from Bioneer Co. (Daejon, South Korea), and annealed by mixing **b** (50 µM, 20 µl), **h** (50 µM, 20 µl), **r** (45 µM, 20 µl), **x** (50 µM, 20 µl), and slowly cooling down from 90°C to the room temperature in 10 mM Tris-HCl (pH 8.0) with 50 mM NaCl.

**b**: Cy5-CCCTAGCAAGCCGCTGCTACGG

**h**: Cy3-CCGTAGCAGCGCGAGCGGTGGG

**r**: biotin-CCCACCGCTCGGCTCAACTGGG-Cy7

**x**: GGGCGGCGACCTCCCAGTTGAGCGCTTGCTAGGG

### Single-molecule experiments

A sample chamber was made between a cleaned quartz slide and a coverslip using double-sided adhesive tape. DNA molecules were immobilized on quartz surface by successive addition of biotinylated BSA (40 ul, 1 mg/ml, Sigma-Aldrich, ST Louis, MO), streptavidin (0.2 mg/ml, 40 ul, Invitrogen, Carlsbad, CA), and DNA in TN buffer (10 mM Tris-HCl, pH 8.0, 50 mM NaCl). Each addition was incubated for 5 minutes, and followed by washing with TN buffer. The concentration of the DNA solution was adjusted to give a good surface density for single-molecule experiments. After checking that fluorescent spots were well separated from one another, we injected 60 µl of imaging buffer (10 mM Tris-HCl (pH8.0) with 0.4% (w/v) glucose (Sigma-Aldrich), 1% (v/v) Trolox (Sigma-Aldrich), 1 mg/ml glucose oxidase (Sigma-Aldrich), 0.04 mg/ml catalase (Roche, Nutley, NJ)), and designated magnesium ions.

## Results and Discussion

### Determination of three FRET efficiencies in static molecules

**Figure 2:**
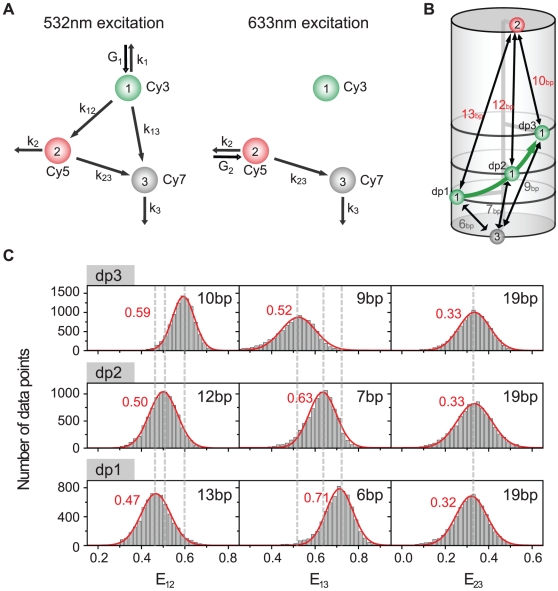
Determination of three FRET efficiencies. (a) An interaction diagram of three dyes upon 532-nm and 633-nm excitations. The identities of symbols used here are explained in the text. (b) Triply-labeled DNA duplexes. The positions of Cy5 and Cy7 are the same, but the labeling position of Cy3 is different in dp1, dp2, dp3. (c) FRET efficiency histograms of the three FRET pairs for each DNA duplex.

Because FRET is a ratiometric method, three fluorescence intensity signals obtained at one excitation condition are not enough to determine the three inter-dye distances. To get an additional relation, we used fast switching of two excitation lasers of 532-nm and 633-nm. The use of two excitation lasers in three-color FRET is simple as well as economical compared to the conventional three-color ALEX technique based on three excitation lasers [9]. However, the capability of determining three FRET efficiencies in real time is not hampered. Figure 2a shows the interaction diagrams of three dyes at each excitation condition. The identities of symbols used here include: G_i_, the excitation rate of the i^th^ dye; k_i_, intrinsic decay rate of the i^th^ dye; k_ij_, the rate of FRET from the i^th^ dye to the j^th^ dye; R_0,ij_, the Förster distance of the corresponding FRET pair; R_ij_, the distance between the i^th^ dye to the j^th^ dye. In this notation, the FRET efficiency from the i^th^ dye to the j^th^ dye is expressed as follows.
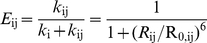
(13)


It is important to notice that E_ij_ is the FRET efficiency expected when only the i^th^ dye and the j^th^ dye exist. So, in the presence of all three dyes, it cannot be calculated simply from the fluorescence intensities of the two dyes as I_j_/(I_i_+I_j_) because fluorescence intensities of the two dyes can be affected by the presence of the other dye.

From rate equations, the gamma corrected fluorescence intensities of the three fluorophores upon 532-nm excitation are written as follows.
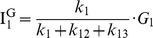
(14)

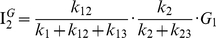
(15)

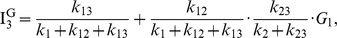
(16)where 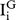 is the gamma-corrected fluorescence intensity of the i^th^ dye after whole correction steps when excited by the green laser. In rigorous forms, the quantum yield and the detection efficiency of the first dye should be multiplied to the right side of the above equations, but these common proportional factors are omitted here for simplicity.

Upon 633-nm excitation, the gamma-corrected fluorescence intensities of the second and the third dyes are written as follows.
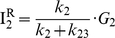
(17)

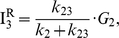
(18)where 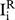 is the gamma-corrected fluorescence intensity of the i^th^ dye after whole correction steps when excited by the red laser. For the same reason as above, the common proportional factors were not written here.

By solving Eqs. 14–18, we obtain E_ij_'s as follows.
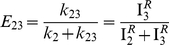
(19)

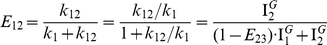
(20)

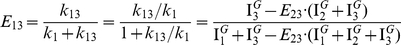
(21)


To test the reliability and the accuracy of our FRET calculation scheme, we used triply-labeled DNA duplexes (Figure 2b). The three DNA duplexes have the same labeling positions of Cy5 and Cy7, but a different position of Cy3 in-between so that the distance between Cy3 and Cy7 increases in the order of dp1, dp2, and dp3. And Figure 2c shows FRET histograms obtained by analyzing more than 60 immobilized molecules, which are qualitatively consistent with the expectation based on sample design; the FRET efficiency of the Cy3-Cy5 pair increases in the order of dp1, dp2, and dp3, and the FRET efficiency of the Cy3-Cy7 pair decreases in the same order, but that of the Cy5-Cy7 pair remains the same in the error range. It is also remarkable that one base pair difference between dp1 and dp2 is clearly distinguished in the FRET histograms, demonstrating the accuracy of our ALEX single-molecule three-color technique.

### Determination of three FRET efficiencies in dynamic molecules

**Figure 3:**
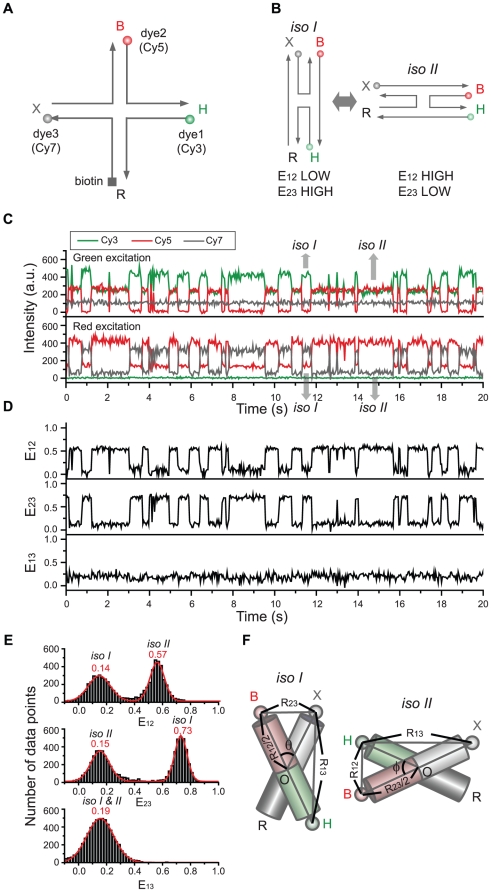
Conformational dynamics of the triply-labeled Holliday junction observed in the confocal setup. (a) Dye labeling scheme of the Holliday junction. (b) Dynamics of the Holliday junction between two conformers, isoI and isoII. (c) Typical fluorescence intensity time traces of Cy3 (green lines), Cy5 (red lines) and Cy7 (gray lines) of the Holliday junction upon 532-nm excitation (upper graphs) or 633-nm excitation (lower graphs). Two excitation lasers were alternatively switched on and off every 20 millisecond synchronously with data binning. The experiments were performed at room temperature in 10 mM Tris-HCl (pH 8.0) with 50 mM Mg^2+^. (d) FRET efficiency time traces calculated from the data in (c). (e) Three inter-dye FRET histograms of the Holliday junction. The histograms were made from more than 20 molecules. (f) Diagram for the determination of inter-duplex angles of isoI and isoII.

The real potential of ALEX single-molecule three-color FRET technique resides in elucidating the complex conformation or reaction dynamics of biomolecules. To demonstrate the capability of our ALEX single-molecule three-color FRET setup, we used a triply-labeled Holliday junction as a model system. As Figure 3a shows, the Holliday junction is labeled with Cy3, Cy5, and Cy7 at the ends of three helical arms, and biotin at the end of the remaining arm. It is known that the Holliday junction switches between two different stacking conformers (isoI and isoII in Figure 3b) [17]–[19]. According to this model, Cy5 is expected to alternatively come close to Cy3 and Cy7 while the distance between Cy3 and Cy7 is maintained.

Figure 3c shows fluorescence intensity time traces of Cy3 (green lines), Cy5 (red lines), and Cy7 (gray lines) from a representative single Holliday junction upon 532-nm excitation (upper graphs) or 632-nm excitation (lower graphs). We used 20-ms bin time, and two excitation lasers were alternatively switched on and off synchronously with the data acquisition. Because the data is so clear, we can tell without any rigorous analysis the anti-correlated behavior of E_12_ and E_23_. To get quantitative understanding, however, we need to perform the FRET analysis as in Eqs. 19–21. Figure 3d shows FRET efficiency time traces calculated from the fluorescence intensity time traces of the molecule in Figure 3c. Consistently with the expectation that the Holliday junction switches between two different stacking conformers, isoI and isoII, E_12_ and E_23_ change with clear anti-correlation, while E_13_ remains the same for the entire observation time. The same conclusion was reached by using a different labeling scheme (Figure S1), confirming that our calculation of multiple FRET efficiencies does not depend on dye labeling positions, but only on molecular conformation. Dwell-time histograms of triply-labeled Holliday junctions gave the similar transition times as in the previously reported two-color FRET experiments [18], confirming that labeling of three dyes does not hinder the intrinsic conformational dynamics of the Holliday junction (Figure S2). A few data points without anticorrelation of E_12_ and E_23_ in Figure 3c, d are due to the Holliday junction dynamics faster than laser switching time.

Simultaneous determination of multiple FRET efficiencies in dynamic molecules suggests that it may be possible to determine the inter-duplex angles of the Holliday junction in two isoforms on the condition that Förster distances of three FRET pairs are accurately known. To do this, we made the FRET histograms of E_12_, E_23_, and E_13_ from ∼20 molecules (Figure 3e), and from the fitting of the histograms to Gaussian distributions, we obtained the center position of E_12_, E_23_, and E_13_ as 0.14, 0.73, and 0.19 for the isoI, and 0.57, 0.15, and 0.19 for isoII. By using the R_0_ values of the three FRET pairs, we obtained the inter-dye distances, R_12_, R_13_ and R_23_, as 7.3nm, 4.8nm and 5.3nm for isoI and 5.2nm, 4.8nm and 8.3nm for isoII. And, finally, the inter-duplex angles in the two isoforms, θ and Φ, were calculated by using the Pappus's centroid theorem (Figure 3f), and they were 95° and 94° for the isoI and isoII, respectively. However, the above calculation was based on an assumption that the inter-dye angles are random, which might be wrong; it was recently reported that the stacking of cyanine dyes at the end of DNA duplex greatly affect the κ^2^ values, and thus the Förster distances [20]. Therefore, it may not be surprising that there is a big discrepancy between the above calculation, and the previously reported value of ∼40° in the crystal structure [21] or ∼60° of the ensemble FRET study [22]. However, it is still possible that the discrepancy originates from DNA deformation during crystallization. With more accurate estimation of κ^2^ values, we will be able to correctly determine inter-helical angles.

### ALEX three-color FRET in TIRF microscopy

**Figure 4:**
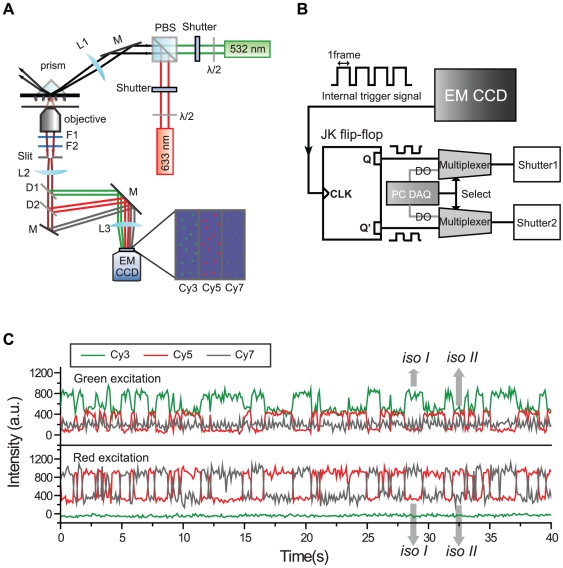
Conformational dynamics of the triply-labeled Holliday junction observed in the TIRF setup. (a) A schematic diagram of ALEX three-color FRET setup in TIRF microscopy. Then setup was built based on an inverted microscope (IX71, Olympus). For switching purposes, mechanical shutters (LS-3, Uniblitz, Rochester, NY) were used. The fluorescence signals of the three dyes were collected by a water-immersion objective (UPlanSApo 60×, Olympus) and focused on different areas of an electron-multiflier charge coupled device (EM-CCD, iXon^+^ DU897BV, Andor Technology, Belfast, UK). The identities of other optics are: F1, notch filter (NF03-633E-25, Semrock, Rochester, NY); F2, long-pass filter (LP03-532RU-25, Semrock); D1, dichroic mirror (635dcxr, Chroma); D2, dichroic mirror (740dcxr, Chroma); L1, lens (LA1986-A, Thorlabs); L2, lens (LAO-120.0-40.0/066, CVI); L3, lens (LAO-260.1-50.0/066, CVI); M, mirror (BB01-E02, Thorlabs). (b) A circuit diagram for synchronization of laser switching and image acquisition. The internal trigger line of the EM-CCD is connected to the clock line of a JK flip-flop (74LS112, Motorola, Schaumburg, IL), and the JK flip-flop generates two complementary pulse trains, Q and Q′, which are used to alternately switch two excitation lasers synchronously. A multiplexer is used to select either a data acquisition board (DAQ PCI-6503, NI) or EM-CCD as a source of shutter control signal. (c) Fluorescence intensity time traces of the Holliday junction observed in TIRF microscope with 50-ms bin time. Experiments were performed at room temperature in 10 mM Tris-HCl (pH 8.0) with 200 mM Mg^2+^.

Compared to confocal microscopy, TIRF microscopy can be used to observe hundreds of single-molecules at a time [2]. This is a huge advantage when the molecular interaction is irreversible. In this work, we also realized an ALEX single-molecule three-color FRET setup based on a conventional prism-type TIRF microscope (Figure 4a). Scattered laser lines were filtered out by using a notch filter and a long-pass filter, and fluorescence signals of three dyes were separated by two dichroic mirrors, and focused on different areas of the EM-CCD camera. The details of the optics and instruments are described in the figure caption.

To switch two excitation lasers (532-nm and 640-nm, Compass215M and Cube640-100C, Coherent, Santa Clara, CA), we used mechanical shutters. Relatively slow switching time of a mechanical shutter (∼1 ms) did not cause any practical problem when an electron-multiplying charge-coupled device (EM-CCD) was operated below 30 frames per second. To synchronize the image acquisition and laser switching, we made a simple relay circuit which accepts the internal trigger line of the EM-CCD, or a digital output line of a data acquisition board and output signals to the TTL inputs of a mechanical shutter controller (Figure 4b). The internal trigger line of the EM-CCD camera, which outputs positive pulses during image acquisition, is connected to the clock input of the negative-edge-triggered JK flip-flop. Because the J and K inputs are set to the HIGH state, the two output signals of the JK flip-flop toggle in a complementary way at every negative edge of the input pulses. Multiplexers were used to switch between an automatic control mode and a manual control mode. Typical fluorescence intensity time traces of the Holliday junction observed in the TIRF setup with 50-ms time resolution are shown in Figure 4c. Although the signal-to-noise ratio of TIRF setup is a little bit lower than that of confocal setup, possibly due to non-negligible readout noise of EM-CCD camera at high speed mode and low quantum yield of the camera at near-infrared region, it is much better than the previously reported ones, demonstrating that the ALEX three-color single-molecule FRET technique can be reliably operated in TIRF microscope, too. In addition to that, our scheme of ALEX three-color FRET technique based on Cy7 provides several times longer observation time compared to the technique based on Alexa 488, or its alternatives (Figure S3).

### Summary

We developed a new ALEX single-molecule three-color FRET setup based on Cy3, Cy5, and Cy7. Thanks to the superior photostability of these dyes, we could obtain a long observation time of immobilized molecules. Negligible spectral overlap of fluorophores enabled us to realize the ALEX technique both in confocal microscopy and in TIRF microscopy, thus providing a way to calculate all FRET efficiencies of the three FRET pairs of dynamic molecules in real time.

## Supporting Information

Figure S1 The conformational dynamics of the Holliday junction with a different labeling scheme. (a) Dye labeling scheme. (b) Conformational dynamics between two isoforms. (c) Fluorescence intensity time traces of the Holliday junction upon 532-nm excitation (upper graphs), and 633-nm excitation (lower graphs). The experimental condition is the same as in Fig. 3. (d) FRET efficiency time traces calculated from (c).
(0.22 MB PDF)

Figure S2 Transition rates of the Holliday junction at 20 °C with 50 mM MgCl2. The dwell-time histograms of isoI (a), and isoII (b) were made from more than 30 molecules with all three dyes, and fitted to single-exponential functions, and their transition times were obtained as 0.22 s, and 0.24 s, respectively. These numbers are little bit larger than the numbers previously reported by McKinney et al. on Nat. Struct. Biol. in 2003 (0.18 s, and 0.16 s, respectively). However, considering that they did experiments at 25 °C, our results are consistent with theirs.
(0.04 MB PDF)

Figure S3 Photobleaching time of Alexa 488, Cy5 and Cy7. (a) Typical intensity time traces of the Holliday junction labeled with Alexa488 and Cy3. (b) Histogram of Alexa488 photobleaching time. The histogram was fitted to a single-exponential curve, and 43 s of photobleaching time was obtained. (c), and (e) Representative intensity time traces of the Holliday junction labeled with Cy3, Cy5 and Cy7. During 300 s observation time, 64% of molecules showed Cy5 photobleaching first as in (c), and 22% of molecules showed Cy7 photobleaching first as in (e). The rest of molecules didn't show any photobleaching of either Cy5 nor Cy7. (d) Histogram of Cy5 photobleaching time with 157-s decay constant. (f) Histogram of Cy7 photobleaching time with 123-s decay constant.
(0.19 MB PDF)

## References

[1] Ha T, Enderle T, Ogletree DF, Chemla DS, Selvin PR (1996). Probing the interaction between two single molecules: fluorescence resonance energy transfer between a single donor and a single acceptor.. Proc Natl Acad Sci U S A.

[2] Ha T (2001). Single-molecule fluorescence resonance energy transfer.. Methods.

[3] Hohng S, Zhou R, Nahas MK, Yu J, Schulten K (2007). Fluorescence-force spectroscopy maps two-dimensional reaction landscape of the Holliday junction.. Science.

[4] Tarsa PB, Brau RR, Barch M, Ferrer JM, Freyzon Y (2007). Detecting force-induced molecular transitions with fluorescence resonant energy transfer.. Angew Chem Int Ed.

[5] Shroff H, Reinhard BM, Siu M, Agarwal H, Spakowitz A (2005). Biocompatible force sensor with optical readout and dimensions of 6nm^3^.. Nano Lett.

[6] Lee M, Kim SH, Hong S (2010). Minute negative superhelicity is sufficient to induce the B-Z transition in the presence of low tension.. Proc Natl Acad Sci U S A.

[7] Hohng S, Joo C, Ha T (2004). Single-molecule three-color FRET.. Biophys J.

[8] Clamme JP, Deniz AA (2005). Three-color single-molecule fluorescence resonance energy transfer.. Chem Phys Chem.

[9] Lee NK, Kapanidis AN, Koh HR, Korlann Y, Ho SO (2007). Three-color Alternating-Laser Excitation of single molecules: monitoring multiple interactions and distances.. Biophys J.

[10] Ross J, Buschkamp P, Fetting D, Donnermeyer A, Roth CM (2007). Multicolor single-molecule spectroscopy with alternating laser excitation for investigation of interactions and dynamics.. J Phys Chem B.

[11] Kapanidis AN, Lee NK, Laurence TA, Doose S, Margeat E (2004). Fluorescence-aided molecule sorting: analysis of structure and interactions by alternating-laser excitation of single molecules.. Proc Natl Acad Sci U S A.

[12] Margeat E, Kapanidis AN, Tinnefeld P, Wang Y, Mukhopadhyay J (2006). Direct observation of abortive initiation and promoter escape within single immobilized transcription complexes.. Biophys J.

[13] Aitken CE, Marshall RA, Puglisi JD (2008). An Oxygen scavenging system for improvement of dye stability in single-molecule fluorescence experiments.. Biophys J.

[14] Roy R, Hohng S, Ha T (2008). A practical guide to single-molecule FRET.. Nat Methods.

[15] Rasnik I, McKinney SA, Ha T (2006). Nonblinking and long-lasting single-molecule fluorescence imaging.. Nat Methods.

[16] Clegg RM (1992). Fluorescence resonance energy transfer and nucleic acids.. Methods Enzymol.

[17] Duckett DR, Murchie AI, Diekmann S, Kitzing E, Kemper B (1988). The structure of the Holliday junction, and its resolution.. Cell.

[18] McKinney SA, Déclais AC, Lilley DMJ, Ha T (2003). Structural dynamics of individual Holliday junctions.. Nat Struct Biol.

[19] Joo C, McKinney SA, Lilley DMJ, Ha T (2004). Exploring rare conformational species and ionic effects in DNA Holliday junctions using single-molecule spectroscopy.. J Mol Biol.

[20] Iqbal A, Arslan S, Okumus B, Wilson TJ, Giraud G (2008). Orientation dependence in fluorescent energy transfer between Cy3 and Cy5 terminally attached to double-stranded nucleic acids.. Proc Natl Acad Sci U S A.

[21] Ortiz-Lombardia M, Gonzalez A, Eritja R, Aymami J, Azorin F (1999). Crystal structure of a DNA Holliday junction.. Nat Struct Biol.

[22] Murchie AI, Clegg RM, Kitzing E, Duckett DR, Diekmann S, Lilley DMJ (1989). Fluorescence energy transfer shows that four-way DNA junction is a right-handed cross of antiparallel molecules.. Nature.

