# Supplementary figures and images for: Single-Molecule Three-Color FRET with Both Negligible Spectral Overlap and Long Observation Time
Source: PLoS One. 2010 Aug 19;5(8):e12270. doi: 10.1371/journal.pone.0012270 (PMC2924373; doi:10.1371/journal.pone.0012270)

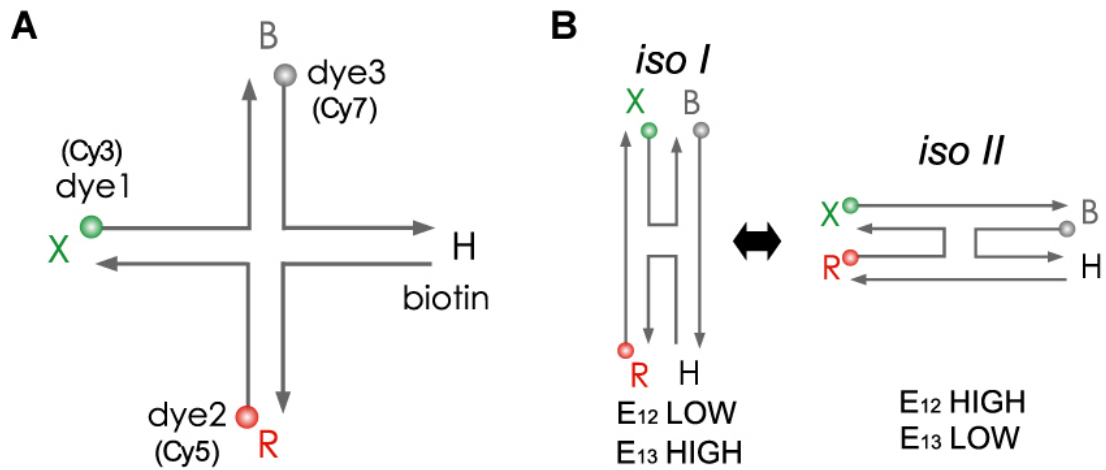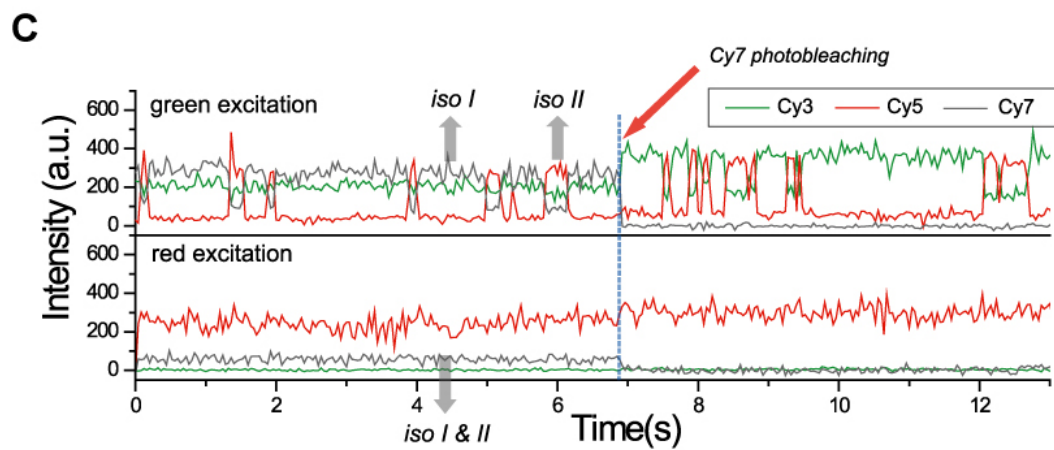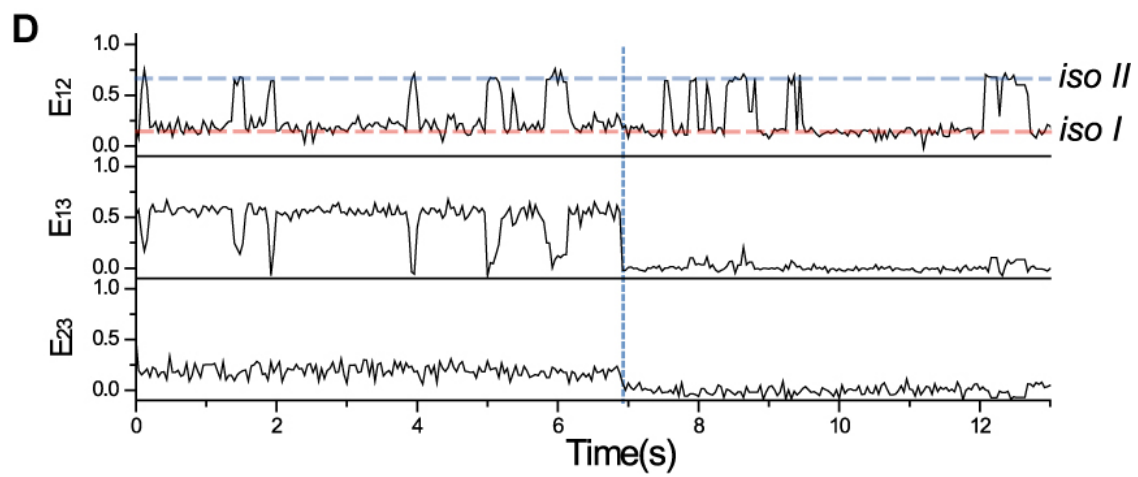

Supplement: Figure S1 — The conformational dynamics of the Holliday junction with a different labeling scheme. (a) Dye labeling scheme. (b) Conformational dynamics between two isoforms. (c) Fluorescence intensity time traces of the Holliday junction upon 532-nm excitation (upper graphs), and 633-nm excitation (lower graphs). The experimental condition is the same as in Fig. 3. (d) FRET efficiency time traces calculated from (c). (0.22 MB PDF) [file pone.0012270.s001.pdf]

**A**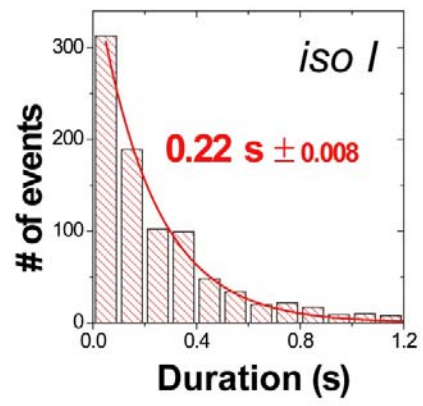**B**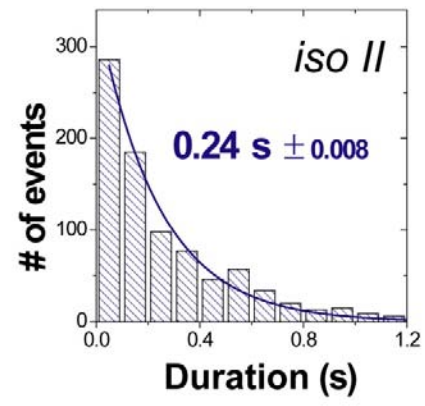

Supplement: Figure S2 — Transition rates of the Holliday junction at 20 °C with 50 mM MgCl2. The dwell-time histograms of isoI (a), and isoII (b) were made from more than 30 molecules with all three dyes, and fitted to single-exponential functions, and their transition times were obtained as 0.22 s, and 0.24 s, respectively. These numbers are little bit larger than the numbers previously reported by McKinney et al. on Nat. Struct. Biol. in 2003 (0.18 s, and 0.16 s, respectively). However, considering that they did experiments at 25 °C, our results are consistent with theirs. (0.04 MB PDF) [file pone.0012270.s002.pdf]

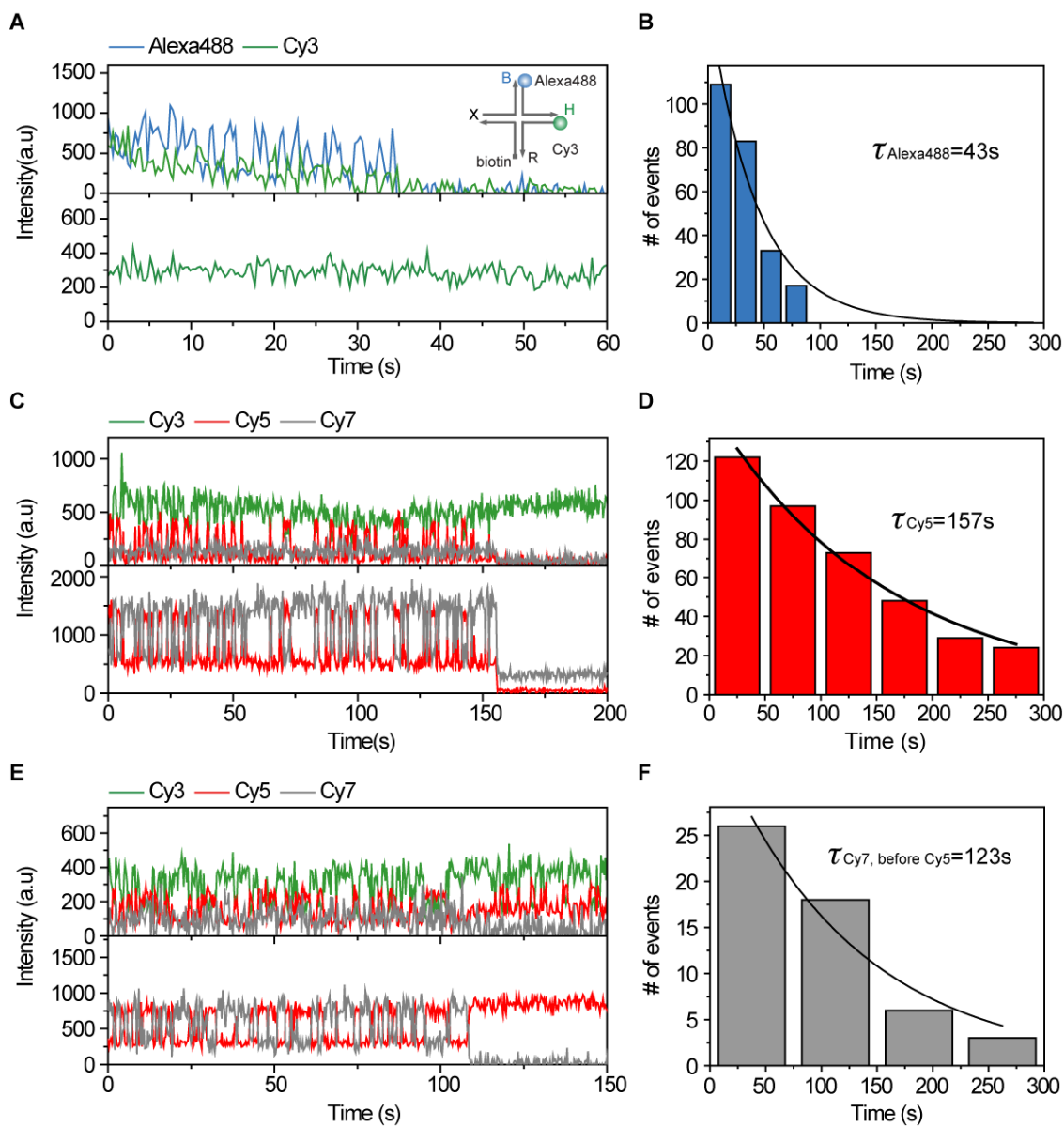

Supplement: Figure S3 — Photobleaching time of Alexa 488, Cy5 and Cy7. (a) Typical intensity time traces of the Holliday junction labeled with Alexa488 and Cy3. (b) Histogram of Alexa488 photobleaching time. The histogram was fitted to a single-exponential curve, and 43 s of photobleaching time was obtained. (c), and (e) Representative intensity time traces of the Holliday junction labeled with Cy3, Cy5 and Cy7. During 300 s observation time, 64% of molecules showed Cy5 photobleaching first as in (c), and 22% of molecules showed Cy7 photobleaching first as in (e). The rest of molecules didn't show any photobleaching of either Cy5 nor Cy7. (d) Histogram of Cy5 photobleaching time with 157-s decay constant. (f) Histogram of Cy7 photobleaching time with 123-s decay constant. (0.19 MB PDF) [file pone.0012270.s003.pdf]
